# Humoral, Cellular and Cytokine Immune Responses Against SARS-CoV-2 Variants in COVID-19 Convalescent and Confirmed Patients With Different Disease Severities

**DOI:** 10.3389/fcimb.2022.862656

**Published:** 2022-05-17

**Authors:** Chun-Hsiang Chiu, Yu-Hsiu Chang, Feng-Yee Chang, Yi-Jen Hung, Ching-Len Liao, Kuo-Chou Chiu, Pei-Ling Tsai, Tien-Wei Chang, Li-Chen Yen

**Affiliations:** ^1^ Division of Infectious Diseases and Tropical Medicine, Department of Internal Medicine, Tri-Service General Hospital, National Defense Medical Center, Taipei, Taiwan; ^2^ Institute of Preventive Medicine, National Defense Medical Center, Taipei, Taiwan; ^3^ National Mosquito-Borne Diseases Control Research Center, National Health Research Institute, Miaoli, Taiwan; ^4^ Department of Microbiology and Immunology, National Defense Medical Center, Taipei, Taiwan; ^5^ Department of Family Dentistry, Tri-Service General Hospital, National Defense Medical Center, Taipei, Taiwan; ^6^ School of Dentistry, National Defense Medical Center, Taipei, Taiwan

**Keywords:** SARS-CoV-2 variants, humoral immune response, cellular immune response, disease severity, inflammatory mediators

## Abstract

**Objectives:**

To assess humoral and cellular immune responses against SARS-CoV-2 variants in COVID-19 convalescent and confirmed patients, to explore the correlation between disease severity, humoral immunity, and cytokines/chemokines in confirmed patients, and to evaluate the ADE risk of SARS-CoV-2.

**Methods:**

Anti-RBD IgG were quantified using an ELISA. Neutralization potency was measured using pseudovirus and real virus. Cellular immunity was measured using ELISpot. Cytokine/chemokine levels were detected using multiplex immunoassays. *In vitro* ADE assays were performed using Raji cells.

**Results:**

One-month alpha convalescents exhibited spike-specific antibodies and T cells for alpha and delta variants. Notably, the RBD-specific IgG towards the delta variant decreased by 2.5-fold compared to the alpha variant. Besides, serum from individuals recently experienced COVID-19 showed suboptimal neutralizing activity against the delta and omicron variants. Humoral immune response, IL-6, IP-10 and MCP-1 levels were greater in patients with severe disease. Moreover, neither SARS-CoV-1 nor SARS-CoV-2 convalescent sera significantly enhanced SARS-CoV-2 pseudovirus infection.

**Conclusions:**

Significant resistance of the delta and omicron variants to the humoral immune response generated by individuals who recently experienced COVID-19. Furthermore, there was a significant correlation among disease severity, humoral immune response, and specific cytokines/chemokine levels. No evident ADE was observed for SARS-CoV-2.

## Introduction

Coronavirus disease 2019 (COVID-19) caused by severe acute respiratory syndrome coronavirus 2 (SARS-CoV-2) has led to a large number of infections and deaths (Hu et al., 2021). Several variants known as variants of concern (VOCs) such as the delta and omicron variants had shown superior infectivity and even immune evasion ability (Harvey et al., 2021). Recent studies have reported that serum from recovered patients previously infected with the Wuhan strain showed a dramatic 10-fold decrease in the neutralizing efficacy against the omicron variant compared to the original strain with D614G mutation (Ma et al., 2022), and another study also indicated that no cross-neutralization towards the omicron variant was observed in unvaccinated alpha variant convalescent patients (Rössler et al., 2022). Cellular immunity is important in combating viral infections, which may provide protective immunity and limit severe disease, and it is important to assess whether individuals who have experienced SARS-CoV-2 infection are able to produce effective cellular immune memory against emerging variants (Rydyznski Moderbacher et al., 2020; Moss, 2022). In addition, some severe COVID-19 patients could have higher antibody levels and neutralizing titers, together with excess proinflammatory cytokine and chemokine levels (Fajgenbaum and June, 2020; Ling et al., 2021; Pum et al., 2021); however, the correlation between these indicators and COVID-19 disease severity is still unclear. Furthermore, previous studies have reported that antibodies against the SARS-CoV-1 spike protein could promote ACE2-independent virus entry into macrophages, monocytes, and B cells *in vitro* (Jaume et al., 2011; Wang et al., 2014; Yip et al., 2014). Whether antisera elicited by previous SARS-CoV-1 or SARS-CoV-2 infection could induce antibody-dependent enhancement (ADE) of SARS-CoV-2 entry is still unknown. In this study, we aimed to investigate the humoral and cellular immune responses from alpha variant convalescent and confirmed patients against emerging SARS-CoV-2 variants. Next, we analyzed the differences in humoral immune responses and cytokine/chemokine profiles in patients with mild/severe disease severity. Finally, we explored whether SARS-CoV-1 and SARS-CoV-2 convalescent sera would enhance SARS-CoV-2 pseudovirus variant infection in Fcγ receptor (FcγR)-expressing Raji cells.

## Materials and Methods

### Cells Lines and Viruses

BHK-21 cells, a baby hamster kidney cell line (ATCC CCL-10), were cultured in RPMI 1640 medium containing 5% fetal bovine serum (FBS; HyClone). HEK293 cells, a human embryonic kidney cell line (ATCC CRL-1573), and 293T/17 cells (ATCC CRL-11268) were grown in DMEM containing 10% FBS. Raji cells, a human B lymphocyte cell line (BCRC 60116), were grown in RPMI 1640 containing 10% FBS. Vero E6 cells (an African green monkey kidney cell line, ATCC CRL-1586) were maintained in high-glucose DMEM supplemented with 10% FBS and an antibiotic-antimycotic (Gibco) in a humidified atmosphere of 37°C and 5% CO_2_. The SARS-CoV-2 WA strain (hCoV-19/Taiwan/4/2020) and the two variants of concern (VOCs), namely, B.1.1.7 (hCoV-19/Taiwan/792/2020, alpha variant), and B.1.617.2 (hCoV-19/Taiwan/1144/2021, delta variant), were kindly provided by Taiwan Centers for Disease Control, Ministry of Health and Welfare, and propagated using Vero E6 cells supplemented with 2% FBS. Passage 2 virus was used for all the studies described here. Viral stocks were free of contamination, and viral titers were determined by plaque assay followed by storage of aliquots at −80°C until further use in experiments.

### Participants

A total of 48 consenting patients from the Tri-Service General Hospital (Taiwan) with laboratory-confirmed SARS-CoV-2 infections were enrolled in this study. Among them, samples from 26 unvaccinated individuals infected with the alpha variant were collected one month after discharge from the hospital. In addition, we also analyzed the samples of 22 patients hospitalized between June and July 2021 with confirmed alpha variant infection. Eleven of these patients were categorized as severe patients with pneumonia according to the following definitions: (1) SpO2 <94% without oxygen supply, (2) respiratory frequency >30 breaths/min, and (3) respiratory failure.

### Spike Plasmid Cloning and SARS-CoV-2 Pseudovirus Production

To construct a pseudovirus carrying the spike protein of SARS-CoV-2, the stocks of pseudovirus were produced by co-transfection of luciferase-expressing pLAS3w-FLuc-Ppuro (10 μg) with 2 other plasmids, the pCMV-Δ8.91 (Gag-Pol provider, 6.6 μg) and the following spike plasmids (4.8 μg) to HEK293T cells (4x10^6^ cells per 10-cm dish) by Lipofectamine 3000^®^ transfection reagent (ThermoFisher): pcDNA3.1_spike_del19 (Addgene #155297), pcDNA3.3_CoV2_B.1.1.7 (Addgene #170451), pcDNA3.3_CoV2_501V2 (Addgene #170449), pcDNA3.3-SARS2-B.1.617.2 (Addgene #172320) and SARS-CoV-2 Omicron Strain S gene Human codon_pcDNA3.1(+) (GenScript # MC_0101274). In brief, 64 μL Lipofectamine 3000^®^ transfection reagents were mixed with 500 μL serum free DMEM and sat at the room temperature for 5 minutes then mixed with three DNA plasmids that were diluted in 500 μL serum-free DMEM for another 25 minutes. This DNA- Lipofectamine mixture was then added into each well and incubated at 37°C in a 5% CO_2_ incubator. After overnight incubation in a 37°C, 5% CO_2_ incubator for 18 hours, the transfected cells were replenished with fresh growth media for continuous culture. At 48 hours post-transfection, the pseudovirus containing culture medium was collected by centrifugation at 1,000 x g for 10 minutes to removes unwanted cells or large debris, followed by passing the clarified medium through a 0.45 μm filter (Millipore Corporation. Billerica, MA, USA). Virus can be stored at 4°C for immediate use or frozen at -80°C. The pseudovirus was normalized by a p24 ELISA kit (Takara Bio).

### Generation of Stable BHK-21/ACE2 and HEK293/ACE2 Cells

Overexpression of *ACE2* gene was carried out by infecting cells with Lentivirus. The pLAS3w.Ppuro plasmid was purchased from Academia Sinica RNAi Core (Taipei, Taiwan), and *ACE2* gene constructed into this plasmid named pLAS3w-ACE2-Ppuro. The stocks of Lentiviruses were produced by co-transfection of pLAS3w-ACE2-Ppuro (2.5 μg) with 2 other plasmids, the pCMV-Δ8.91 (Gag-Pol provider, 2.25 μg) and the pMD2.G (VSV-G pleotropic envelope provider, 0.25 μg), to HEK293T cells (1.5x10^6^ cells per 6-cm dish) by Lipofectamine 3000^®^ transfection reagent (ThermoFisher). In brief, 15 μL Lipofectamine 3000^®^ transfection reagents were mixed with 250 μL serum free DMEM and sat at the room temperature for 5 minutes then mixed with three DNA plasmids that were diluted in 250 μL serum-free DMEM for another 25 minutes. This DNA- Lipofectamine mixture was then added into each well and incubated at 37°C in a 5% CO2 incubator. After overnight incubation in a 37°C, 5% CO_2_ incubator for 18 hours, the transfected cells were replenished with fresh growth media for continuous culture. At 48 hours post-transfection, the Lentivirus containing culture medium was collected by centrifugation at 1,000 x g for 10 minutes to removes unwanted cells or large debris, followed by passing the clarified medium through a 0.45 μm filter (Millipore Corporation. Billerica, MA, USA). For ACE2 overexpression, BHK-21 or HEK293 cells were seeded at a density of 1.5x10^6^ cells per well in 6 cm dishes. After overnight culture, cells were incubated with lentivirus containing 8 μg/mL polybrene in 1 mL of fresh growth medium for 1-hour viral absorption at 37°C in a 5% CO_2_ incubator with gently rocking per 15 minutes. After the absorption process, 3 mL growth medium containing 8 μg/ml polybrene were supplemented for continuous cell cultivation for 24 hours. The following days, puromycin was added to the culturing medium at a concentration of 10 μg/mL for at least 48 hours to select for survived ACE2-overexpressing cell clones.

### Western Blot

To investigate the ACE2 expression of BHK-21/ACE2 cells and HEK293/ACE2 cells, the cells were dissolved with RIPA buffer. The cell lysates were analyzed by western blot analysis with the anti-ACE2 antibody (GeneTex 101395) and anti-actin antibody (Millipore). Then, membranes were probed with the secondary antibody horseradish peroxidase-conjugated goat anti-mouse IgG antibody (Jackson ImmunoResearch). The signals were developed by enhanced chemiluminescence (Millipore) and photographed by using Luminescent Image Analyzer (LAS-3000; Fujifilm).

### RBD-Specific IgG ELISA

To quantitatively detect IgG antibodies against the SARS-CoV-2 RBD, we used an indirect ELISA using an anti-SARS-CoV-2 IgG1 monoclonal antibody (CR3022) (Yuan et al., 2020; Demonbreun et al., 2021; Moriyama et al., 2021; Thomas et al., 2021; Yuen et al., 2021). 96 well ELISA plates (Thermo Fisher) were coated for 16 hours at 4°C with purified SARS-CoV-2 alpha variant RBD (Genetex, Cat No. GTX136014-pro) or delta variant RBD (GeneTex, Cat. No. GTX136014-pro) diluted in carbonate-bicarbonate buffer to a concentration of 1 μg/mL. The plates were washed with 0.05% PBST, incubated with a blocking buffer consisting of 1% BSA in PBS for 1 hour at room temperature, and then washed. Serum samples were diluted 1:50 with a dilution buffer consisting of 1% BSA. A six-point standard curve was created using CR3022-IgG1 starting at 2 mg/mL by performing 1:2 serial dilutions with dilution buffer (**Supplementary Figure S2**). Samples and standards were added to corresponding wells and incubated for 1 hour at room temperature, followed by washing. Human IgG antibodies were detected with anti-human IgG-HRP (1:100,000). This detection antibody was added to the plate and incubated for 30 minutes at room temperature. After washing, TMB substrate (Invitrogen) was added to each well and incubated for 5 minutes, and the reaction was stopped with 1 M H_2_SO_4_. Optical density (O.D.) was measured at 450 nm with subtraction of the O.D. at 570 nm as a reference wavelength on an ELISA reader (BioTek). Anti-RBD antibody levels were calculated by interpolating onto a standard curve and correcting for sample dilution; one unit per mL (U/mL) was defined as the equivalent reactivity seen with 1 mg/mL CR3022, and the cut-off value was defined as mean OD_450_ values of pre-pandemic sera + 3 SD. All experiments were performed in duplicates.

### Neutralization Assay (NT_50_) With Pseudotyped SARS-CoV-2

BHK-21/ACE2 cells were seeded with 4x10^4^ in 24-well plate at 16 hours before the infection. To test the infectivity of pseudovirus, 100 ng of each variant of pseudovirus were add to BHK-21/ACE2 cells and incubated for 48 hours. For neutralization assay, 50 μl of heat-inactivated sera were 2-fold diluted in duplicate samples in complete medium with 2% FBS starting with a 1:16 dilution followed by incubation with 50 μl of pseudovirus (1 ng p24) for 1 h at 37°C. On the day of infection, the cells were washed twice with PBS, and 100 μl of inoculum was added to the cells and incubated for 48 hours. The cells were quenched by adding 100 μl of BrightGlow luciferase substrate (Promega) directly to each well, and the luciferase activity was measured with Synergy H4 luminometer (BioTek). Background values, monitored from uninfected cells were consistently below 400 relative luminescence units, and sera collected before 2019 were used to set as the negative control for the neutralization assay, sera started diluted at 1:16, gave results in the range of the background RLU levels. An NT_50_ > 1:16 serum dilution was regarded as positive.

### Neutralization Assay (PRNT_50_) With Real SARS-CoV-2

Serum samples were heat-inactivated for 30 minutes at 56°C; twofold serial dilutions, starting at a concentration of 1:5, were then mixed with an equal volume of viral solution containing 200 PFU of SARS-CoV-2. The serum-virus mixture was incubated for 1 hour at 37°C in a humidified atmosphere with 5% CO_2_. After incubation, the mixture at each dilution was added to Vero E6 cells and incubated at 37°C for 1 hour. Cells were subsequently cultured with DMEM containing 2% FBS and 1.4% methylcellulose for 72 hours. After culturing, plaques were stained and counted. Neutralizing antibody titers were defined as the reciprocal of the maximum dilution of serum that reduced the virus titer by 50% compared to the negative control sera, and PRNT_50_ below 1:5 serum dilution was considered negative.

### Isolation of PBMCs

PBMCs of COVID-19 convalescent individuals were isolated from anticoagulant blood using Ficoll-PaqueTM PLUS density gradient medium (Cytiva #17144003). To isolate PBMCs, blood diluted with PBS was gently layered over an equal volume of Ficoll in a Falcon tube and centrifuged for 30 minutes at 400 x g without braking. Four layers formed, each containing different cell types. The second layer contained PBMCs. These cells were gently removed using a Pasteur pipette and added to warm medium or PBS to wash off any remaining platelets. The pelleted cells were then counted, and the percentage viability was estimated using Trypan blue staining. Isolated PBMCs were stored in liquid nitrogen until use in assays.

### ELISpot Assay

The number of antigen-specific IFN-γ- or IL-2-secreting SFU was determined by ELISpot assays. Cryopreserved PBMCs were rapidly thawed and allowed to rest overnight. Cells were dispensed at 1 × 10^5^ cells per well for the IFN-γ or IL-2 ELISpot assay (Human IFN-γ or IL-2 ELISpot Kit, R&D Systems). The cells were stimulated with a pool of peptides consisting mainly of 15-mer sequences with 11 amino acid overlap, covering the S protein selectively mutated regions of the SARS-CoV-2 alpha variant (PepTivator^®^ SARS-CoV-2 Prot_S B.1.1.7, Miltenyi Biotec) or delta variant (PepTivator^®^ SARS-CoV-2 Prot_S B.1.617.2, Miltenyi Biotec), and incubated at 37°C for 22 hours. Cells stimulated with PHA-M (Phytohemagglutinin, M form, ThermoFisher) served as the positive control and all convalescent patients were above 115 spot forming cells (SFC)/10^5^ PBMCs for both IFN-γ and IL-2. IFN-γ or IL-2 release was detected following the instructions in the manual, and the spots were counted using an ELISPOT reader (Cellular Technology Ltd.). The mean SFC value counted in triplicate peptide pool stimulations was calculated and normalized by subtracting the mean of the negative control replicates (control medium), and the cut-off value for background T cell responses was defined as the mean SFC value of seronegative PBMCs derived from healthy unvaccinated donors + 3 SD (9.2 SFC/10^6^ PBMCs). The results were expressed as SFC per million PBMCs.

### Multi-Plex Immunoassay

To assess cytokines and chemokines concentrations of in confirmed patients, ELISA-based Bio-Plex Pro Human Cytokine, Chemokine, and Growth Factor Assays Kit was used for evaluating the production of IL-6, IP-10 and MCP-1 in sample sera following the manufacturer’s instructions. The patient sera samples were diluted (1:3) in sample diluent and cytokines or chemokines were analyzed with the Bio-Plex200 System using the Bio-Plex Manager™ software. For each cytokine and chemokine, assay ranges and LOD were provided by the manufacturer. All reagents and equipment, including washing station and shaking incubators, were from BIO-RAD Laboratories.

### Antibody-Dependent Viral Entry (*In Vitro* ADE Assay)

For ADE assays, 100 μl of serial 8-fold dilutions of heat-inactivated serum were incubated for 1 hour at 37°C with 100 μl of pseudovirus. One hundred microliters of Raji cells (1×10^6^ cell/mL) previously washed three times with serum-free RPMI were added to the antibody-SARS-CoV-2 pseudovirus mixture in a 96-well plate, and some groups of Raji cells were preincubated with 5 μg/ml FcγR inhibitor (Invitrogen # 16-0329-81) for 15 minutes at 4°C. After adsorption for 24 hours at 37°C, the medium was renewed 24 hours later; the cells were incubated for an additional 24 hours, washed in PBS, and lysed; and luciferase activity was measured with a Synergy H4 luminometer (BioTek). Duplicates were performed for each tested serum and the dotted line represents the average luciferase activity of the virus-only group.

### Ethics

This study was approved by the Tri-Service General Hospital (TSGHIRB No. C202005067). Informed consent was obtained from all enrolled participants.

### Statistical Analysis

Statistics were determined using GraphPad Prism 5. Anti-RBD IgG titers, NT_50_ and PRNT_50_ were described as medians and IQRs. A nonlinear sigmoidal 4PL model was used to determine the NT_50_ and PRNT_50_ for each serum. Measured statistical significance for pseudovirus or real virus neutralization assays (NT_50_ and PRNT_50_) were calculated among experiments by one-way ANOVA with Tukey’s multiple comparison test. Simple linear regression and Pearson correlation analysis were conducted to determine the correlation coefficients between anti-RBD IgG titers and NT_50_ or PRNT_50_. Two-tailed Student’s t test was conducted for indicators of different disease severities. ROC curves were also plotted using GraphPad Prism 5, and the AUC was calculated. Asterisks indicated statistical significance, *p<0.05, **p<0.01, ***p<0.001, ****p<0.0001.

## Results

### Clinical Characteristics of Enrolled Convalescent and Confirmed Patients in This Study

Blood samples were obtained from 26 alpha variant convalescent patients who have been discharged from hospital for 1 month and 22 alpha variant confirmed patients hospitalized between June and July 2021. Among the 26 convalescent patients, 14 (54%) were male and 12 (46%) were female, with a median age of 58 years (54-70 years), while among the 22 confirmed patients, 17 (77%) were male and 5 (23%) were female, with a median age of 56 years (38–68 years). The detailed clinical characteristics of convalescent and confirmed patients are summarized in the **Supplementary Tables 1, 2**.

### Humoral and Cellular Immune Responses of Alpha Variant Convalescent Sera Against Different SARS-CoV-2 Pseudovirus and Real Virus Variants

**Figure 1:**
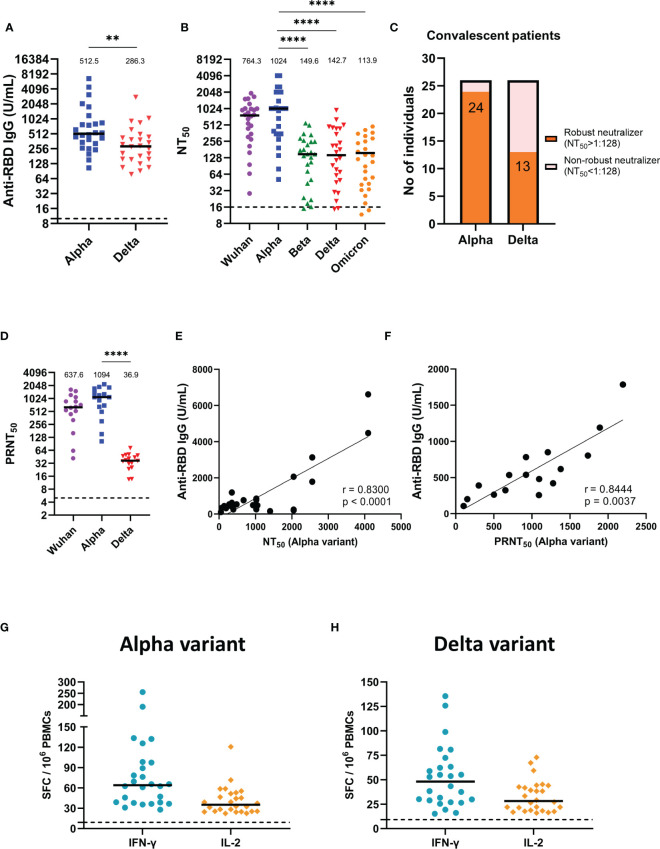
Humoral and cellular immune responses of alpha variant convalescents. **(A)** Results of ELISA measuring serum reactivity to anti-RBD IgG (n = 26). **(B)** NT_50_ of alpha variant convalescent sera (n = 26) measured with indicated SARS-CoV-2 pseudovirus variants. **(C)** Fraction of robust neutralizers in the convalescent cohort at 1-month post-infection. Individuals with a pseudovirus NT_50_ above 1:128 were classified as robust neutralizers, while individuals with a pseudovirus NT_50_ below 1:128 were classified as non-robust neutralizers. **(D)** PRNT_50_ of alpha variant convalescent sera (n = 16) detected with indicated real SARS-CoV-2 variants. **(E)** Correlation between alpha variant anti-RBD IgG titers and NT_50_. **(F)** Correlation between alpha variant anti-RBD IgG titers and PRNT_50_. **(G, H)** IFN-γ and IL-2 ELISpot of alpha variant convalescents PBMCs stimulated with **(G)** alpha variant or **(H)** delta variant peptide pool. SFC, spot-forming cells. The dotted line represents the cut-off value for each assay. Duplicates were performed for each tested sample. Measured statistical significance was calculated among experiments by one-way ANOVA with Tukey’s multiple comparison test. Simple linear regression and Pearson correlation analysis were conducted to determine the correlation coefficients. Asterisks indicate statistical significance, **p < 0.01, ****p < 0.0001.

We first assessed anti-RBD IgG titers in the sera of alpha variant convalescent patients. The results showed that the median (25-75 interquartile range (IQR)) antibody titers against alpha variant was 512.54 U/mL (329.22-837.70). Notably, when faced with the delta variant, which has many mutations in RBD, the median (25-75 IQR) anti-RBD IgG titers was 286.32 U/mL (157.57-435.79), a significant 2.5-fold decrease compared to the alpha variant (p =0.0033) (**Figure 1A**). Next, for the neutralization assay, we established different SARS-CoV-2 pseudovirus variants and ACE2-expression stable cell lines (**Supplementary Figure S1**). Lentiviral packaging plasmid, lentiviral transfer plasmid (with luciferase gene) and SARS-CoV-2-spike plasmid with indicated mutation were co-transfection into producer cell (293 cells), after 48 hours transfection, cell supernatant was harvested, purified and the pseudovirus titer was normalized using the p24 ELISA kit (**Supplementary Figure S1A**). After validation of exogenous ACE2 expression in BHK-21 and HEK293 cells by western blot (**Supplementary Figure S1B**), the infectivity assay were performed and showed that pseudovirus variants have significantly higher luciferase activities compared to the Wuhan strain (**Supplementary Figure S1C**), suggesting other variant strains exhibited more efficient ACE2-mediated infection than the wild type Wuhan strain as previously described (Arora et al., 2021; Kuzmina et al., 2021; Hoffmann et al., 2022). Then, we used this platform to evaluate the neutralizing abilities of alpha variant convalescent sera against different SARS-CoV-2 pseudovirus variants (**Figure 1B**). The results demonstrated that alpha variant convalescent patients showed the greatest neutralization efficacy (NT_50_) for the alpha strains (**Figure 1B**, blue). However, the NT_50_ against the beta, delta and omicron variants decreased significantly, with a 5.2-fold reduction for the delta variant and a 7.7-fold reduction for the omicron variant compared to that for the alpha variant (p < 0.0001). We then further divided these convalescent individuals into robust neutralizers (serum diluted 1:128 could still neutralize more than half of the pseudovirus) and non-robust neutralizers (**Figure 1C**) (Planas et al., 2021). Most of the sera from the alpha convalescent individuals (24/26) robustly neutralized the alpha variant, compared to the delta variant, for which only 50% of the convalescent sera (13/26) effectively neutralized the pseudovirus. Likewise, a similar trend was found by using real SARS-CoV-2, with a remarkable decline in PRNT_50_ for the delta variant compared to that for the alpha variant (p < 0.0001) (**Figure 1D**). These results suggested that the beta, delta and omicron variants are more resistant to alpha convalescent serum neutralization. Furthermore, we correlated anti-RBD IgG titers with NT_50_ and PRNT_50_ measured by using pseudovirus (**Figure 1E**) and real SARS-CoV-2 assays (**Figure 1F**). The results showed a significant positive correlation between the anti-RBD IgG titers and the corresponding NT_50_ or PRNT_50_ in alpha variant convalescent patients, indicating that anti-RBD IgG is crucial for neutralizing SARS-CoV-2. Subsequently, we also assessed the cellular immune memory of T cells after stimulation by alpha and delta variants spike peptide pools using IFN-γ and IL-2 ELISpot assays (**Figures 1G, H**). The results showed that for alpha and delta variants the median (25-75 IQR) number of IFN-γ-secreting T cells were 64 (38.6-95.3) and 48.2(29.1-63) SFC/10^6^ PBMCs, while IL-2 were 35.1 (25.6-50.3) and 28.2 (19.4-42.4) SFC/10^6^ PBMCs, respectively, indicating that alpha variant convalescent patients could produce T cells against alpha and delta variants.

### Neutralizing Potency of the Sera From Alpha Variant Infection-Confirmed Patients Against Different SARS-CoV-2 Pseudovirus and Real Virus Variants

**Figure 2:**
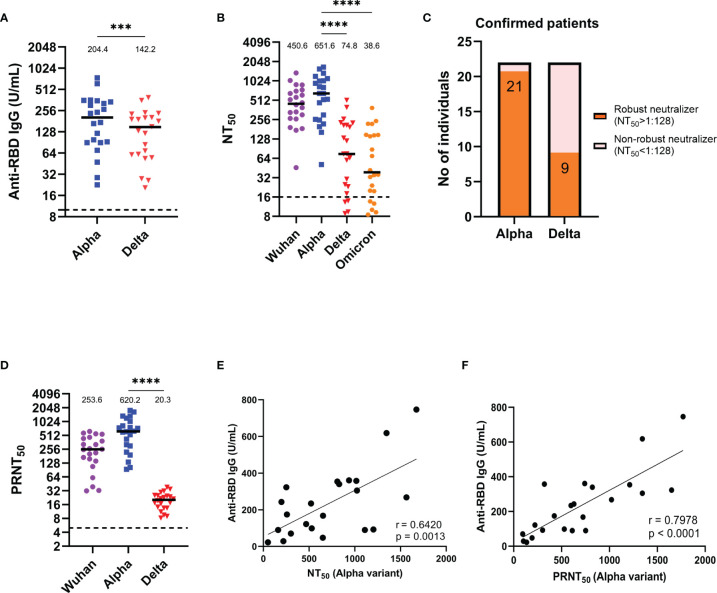
Anti-RBD IgG titers and neutralization abilities of alpha variant infection-confirmed patient serum. **(A)** Results of an ELISA measuring serum reactivity to anti-RBD IgG (n = 22). **(B)** NT_50_ of alpha variant infection-confirmed patients (n = 22) measured with indicated SARS-CoV-2 pseudovirus variants. **(C)** Fraction of robust neutralizers in the alpha variant infection confirmed patient cohort. Individuals with NT_50_ above 1:128 were classified as robust neutralizers, while below 1:128 were classified as non-robust neutralizers. **(D)** PRNT_50_ of alpha variant-confirmed patients (n = 22) measured with the indicated real SARS-CoV-2 variants. **(E)** Correlation between anti-RBD IgG titer and NT_50_. **(F)** Correlation between anti-RBD IgG titer and PRNT_50_. The dotted line represents the cut-off value for each assay. Duplicates were performed for each tested serum. Measured statistical significance was calculated among experiments by one-way ANOVA with Tukey’s multiple comparison test. Simple linear regression and Pearson correlation analysis were conducted to determine the correlation coefficients. Asterisks indicate statistical significance, ***p < 0.001, ****p < 0.0001.

Next, we further measured anti-RBD IgG titers in alpha variant infection-confirmed patients (**Figure 2A**), and the median (25-75 IQR) antibody concentrations in these patients was 204.36 U/mL (90.61-335.60), which is a notably lower titer than that in the one-month alpha convalescent individuals, when faced with delta variant, the anti-RBD IgG titer decreased significantly by 1.6-fold compared to the alpha variant (p = 0.0002) (**Figure 1A**). In addition, the median (25-75 IQR) NT_50_ of these confirmed patients was 450.63 (275.63-692.84) for the Wuhan strain and 651.65 (268.84-1022.09) for the alpha variant (**Figure 2B**). However, the median (25-75 IQR) NT_50_ for the delta variant and omicron variant dropped to 74.80 (23.63-209.03) and 38.64 (19.77-147.09), indicating that the new emerging variants is more resistant to neutralizing antibodies. Additionally, 21 of 22 confirmed patients’ sera (95.5%) were effective in neutralizing the alpha variant, which was reduced to 9 of 22 subjects (40.9%) for the delta variant (**Figure 2C**). Similarly, using real SARS-CoV-2 variants to measure neutralizing activity, we found that the PRNT_50_ for the delta variant decreased 34.6 times compared to that for the alpha variant (p < 0.0001) (**Figure 2D**). Likewise, a significant positive correlation between the anti-RBD IgG titers and the corresponding NT_50_ (**Figure 2E**) or PRNT_50_ (**Figure 2F**) were shown in confirmed patients.

### Discriminating Distinct Characteristics Between Mild and Severe COVID-19 Patients

**Figure 3:**
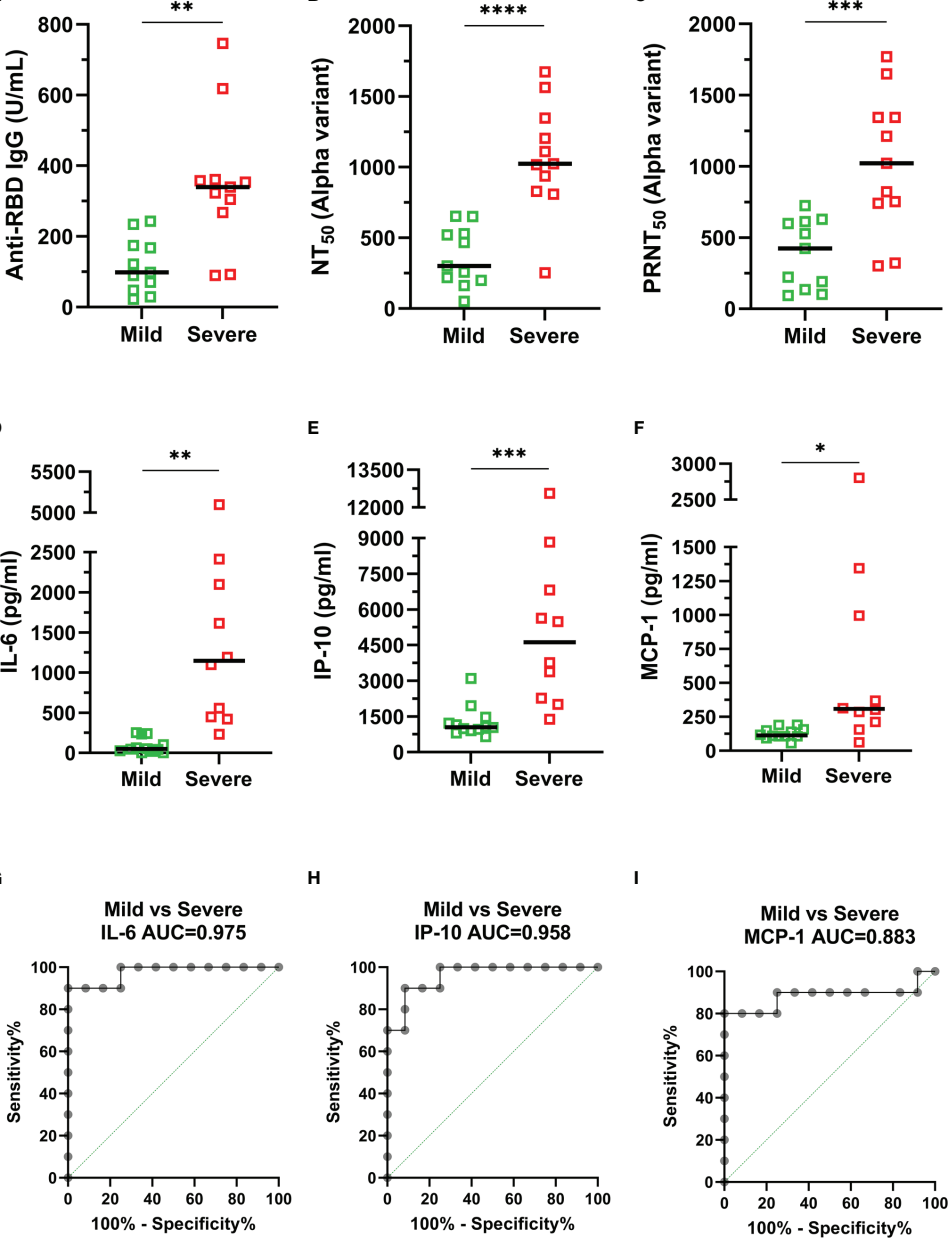
Comparison of relevant indicators between mild (green) and severe (red) COVID-19 confirmed patients. **(A)** Anti-RBD IgG. **(B)** NT_50_ (alpha variant). **(C)** PRNT_50_ (alpha variant). **(D)** IL-6. **(E)** IP-10. **(F)** MCP-1. **(G–I)** The area under the receiver operating characteristic (ROC) curve (AUC) for serum cytokine levels: **(G)** IL-6, **(H)** IP-10, and **(I)** MCP-1. Duplicates were performed for each tested serum. Measured statistical significance was calculated between experiments by two-tailed Student’s t test. Asterisks indicate statistical significance, *p < 0.05, **p < 0.01, ***p < 0.001, ****p < 0.0001.

We further categorized the alpha variant infection-confirmed patients into mild and severe groups. The results revealed that the anti-RBD IgG titers (p = 0.0014), NT_50_ (p < 0.0001) and PRNT_50_ (p = 0.0009) against the alpha variant were significantly higher in the severe group than in the mild group (**Figures 3A–C**). Moreover, IL-6, IP-10, and MCP-1 levels were increased by 16.8- (p = 0.0029), 4.1- (p = 0.0010), and 5.4-fold (p = 0.0331) in patients with severe illness compared to those in patients with mild illness (**Figures 3D–F**). The area under the receiver operating characteristic (ROC) curve (AUC) for serum levels of cytokines and chemokines was used to estimate the likelihood of a patient developing severe disease (**Figures 3G–I**). The results showed that the AUC was 0.975 (95% CI: 0.9175-1.000) for IL-6, followed by 0.958 (95% CI: 0.8842-1.000) for IP-10 and 0.883 (95% CI: 0.8842-1.000) for MCP-1, indicating that these inflammatory indicators have excellent predictive performance for poor prognosis. Taken together, these distinct characteristics between mild and severe patients could be used as predictive markers for disease severity.

### Investigate the ADE Phenomenon by Using SARS-CoV-1 and SARS-CoV-2 Convalescent Sera in FcγR-Expressing Raji Cells

**Figure 4:**
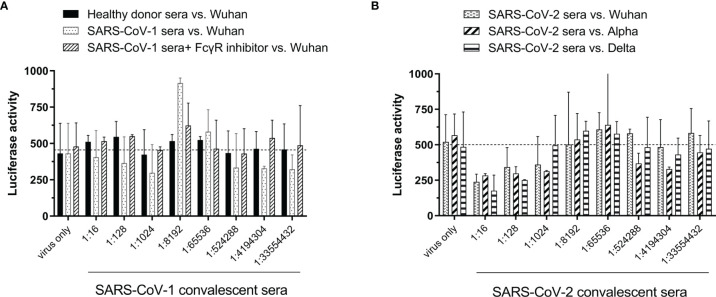
Susceptibility of B lymphoblast Raji cells to infection by SARS-CoV-2 pseudovirus variants under different conditions. **(A)** SARS-CoV-1 convalescent serum vs. SARS-CoV-2 Wuhan strain. **(B)** SARS-CoV-2 alpha variant convalescent serum vs. SARS-CoV-2 Wuhan, alpha or delta strain.

ADE is usually mediated by sub- or non-neutralizing antibodies, and there are currently some variants that are resistant to the neutralizing efficacy of convalescent serum. Therefore, we established an ADE assay by using pseudoviruses and tested the infectivity of SARS-CoV-1 and SARS-CoV-2 alpha variant convalescent sera in Raji cells. As shown in **Figure 4A**, healthy donor sera did not increase the infection of the Wuhan strain compared to that of the virus-only group (dotted line). Although a slight increase in luciferase activity was observed when the SARS-CoV-1 convalescent serum was serially diluted to 1:8192, and this phenomenon could be reduced by the addition of FcγR inhibitor, however, the increment was not significant compared to the virus-only group (**Figure 4A**). In addition, SARS-CoV-2 alpha variant convalescent sera were also examined to determine the ADE phenomenon, but no significant infection enhancement of infection by the Wuhan, alpha, and delta variants was observed (**Figure 4B**).

## Discussion

In this study, we found that individuals who recovered from the alpha variant showed a 2.5-fold decrease in antibodies against delta variant RBD and a dramatically 7.7-fold reduction in neutralizing potency when faced with the emerging omicron variant compared to the alpha variant. Furthermore, only half of the one-month COVID-19 convalescent patients and approximately 40% of the confirmed patients were robust neutralizers (NT_50_ > 1:128) against the delta variant, while all one-month convalescent PBMCs responded to alpha and delta variants spike peptide pools by producing IFN-γ and IL-2. Anti-RBD IgG and neutralizing titers against the alpha variant, as well as levels of pro-inflammatory cytokines (IL-6) and chemokines (IP-10, MCP-1) were significantly higher in the severe group than in the mild group. In addition, an *in vitro* ADE assay showed that both SARS-CoV-1 and SARS-CoV-2 convalescent serum did not significantly enhance the entry of SARS-CoV-2 pseudovirus into FcγR-expressing Raji cells.

Recent studies have reported numerous omicron and delta variants leading to reinfection of individuals who had previously been exposed to SARS-CoV-2 (Pulliam et al., 2021; Shastri et al., 2021) and even breakthrough infection with full vaccination (Goga et al., 2021; Kustin et al., 2021; Kuhlmann et al., 2022). Another study using the pseudotyped SARS-CoV-2 variant omicron to evaluate COVID-19 convalescent patients serum infected with the original strain also showed that compared to the D614G reference strain, the neutralization efficacy of omicron variants decreased approximately 8.4-folds (Wang et al., 2022). Our study also showed that even in newly discharged patients who recently recovered from COVID-19, the neutralizing potency against the delta variant and the omicron variant decreased nearly 5-fold and 7-fold compared to the alpha variant. (**Figure 1B**), suggesting a strong immune escape capability of the delta and omicron variants.

SARS-CoV-2 initiates infection by the interaction between the RBD of the spike protein and the ACE2 receptor. Previous studies have also found a high correlation between RBD-specific IgG levels and neutralizing capacity (Iyer et al., 2020; Wang et al., 2021) and confirmed that neutralizing antibody levels are highly predictive of protection (Khoury et al., 2021). Recent meta-analysis have also indicated that despite the varying degrees of reduction in the neutralizing efficacy of the current vaccines against VOCs compared to the original Wuhan strain, there is still a robust correlation between vaccine-induced neutralizing activity and the protection capability against symptomatic SARS-CoV-2 variants infection (Cromer et al., 2022). In our study, we also found a significant positive correlation between anti-RBD IgG titers and NT_50_ for both pseudovirus (**Figures 1E**, **2E**) and the real virus (**Figures 1F**, **2F**). In the future, as convalescent individuals face a new epidemic of emerging SARS-CoV-2 variants, it will be possible to measure their anti-RBD IgG titers to quickly assess whether they still have sufficient protection against emerging SARS-CoV-2 variants and to serve as a basis for vaccine booster administration.

T cells can activate other immune cells during infection and kill infected cells to control disease progression (Sattler et al., 2020; Le Bert et al., 2021; Moss, 2022). As a previous study revealed that 82 COVID-19 convalescents showed a positive IFN-γ response to the spike peptide pool (Cassaniti et al., 2021), in our study, we also found that all one-month COVID-19 convalescent PBMCs responded to alpha and delta variants spike peptide pool stimulation and produced IFN-γ and IL-2 (**Figures 1G, H**), indicating that these individuals generated cellular immune memory against SARS-CoV-2. However, it is not entirely clear what level of antibodies and/or T cells is necessary to confer such protection against SARS-CoV-2.

Although neutralizing antibodies usually eliminate the virus and provide protection in most viral infections, previous studies have found that both anti-RBD IgG and NT_50_ titers were significantly higher in severe patients than in mild patients (Robbiani et al., 2020; Garcia-Beltran et al., 2021; Lafon et al., 2021; Legros et al., 2021). In our study, we also found that the anti-RBD IgG titer (**Figure 3A**) and neutralization capacity (**Figures 3B, C**) were significantly higher in severe patients, indicating that higher neutralizing antibodies does not appear to protect against COVID-19 progression, and that robust humoral immunities may be a consequence of the exaggerated immune activation in severe SARS-CoV-2 infection (Legros et al., 2021).

Inflammatory mediators are thought to cause severe inflammatory responses to tissue damage, and IL-6, IP-10 and MCP-1 have been found to be associated with acute lung injury and even poor prognosis and higher risk of death in SARS-CoV-1 (Jiang et al., 2005; Chien et al., 2006) and SARS-CoV-2 infections (Chen et al., 2020; Jøntvedt Jørgensen et al., 2020; Liu et al., 2020; Hashimoto et al., 2021). Our results showed that the levels of these three inflammatory factors (IL-6, IP-10, and MCP-1) were indeed significantly higher in the severe group (**Figures 3D–F**). These results not only indicated that measuring the levels of these inflammatory factors could predict the disease severity but also suggested that using inhibitors of these inflammatory factors may be helpful to reduce the risk of death, such as tocilizumab, which targets IL-6R and reduces mortality in severe COVID-19 patients (Somers et al., 2021; Wei et al., 2021). In addition, these cytokines and chemokines, together with other biochemical markers (CRP, D-dimer, and ferritin), may then be used to assess the risk of disease progression in COVID-19 patients during the early stages of the disease and to advance treatment with antiviral or anticytokine/antichemokine drugs to prevent the development of severe illness.

Previous studies have found that anti-spike serum produced by mice immunized with SARS-CoV-1 spike protein can help pseudotyped or real SARS-CoV-1 enter Raji cells *via* the FcγR-dependent pathway (Yip et al., 2014). Thus, ADE is an issue of concern for SARS-CoV-2 because of worries that ADE may lead to more severe forms of the disease in recovered COVID-19 patients or vaccinated individuals (Ricke, 2021). As no infectious virus production were reported after SARS-CoV-1 or SARS-CoV-2 entered Raji cells *via* ADE (Jaume et al., 2011; Zhou et al., 2021), we also found that SARS-CoV-1 convalescent serum slightly enhanced the entry of SARS-CoV-2 pseudovirus (**Figure 4A**), and no significant infection enhancement was observed by using SARS-CoV-2 convalescent sera (**Figure 4B**). Overall, these results suggested that SARS-CoV-2 infection may not induce viral replication enhancement by ADE.

The limitations of the study include the small sample size that may not represent neutralizing potency in diverse populations, in addition to the lack of serial samples and longitudinal assessments. We will continue to follow participants and evaluate their humoral and cellular immune responses against emerging variants after subsequent vaccination. Moreover, the phenomenon observed in the ADE assay using the FcγR-expressing cell line does not necessarily mean that the same situation will be observed *in vivo*. Recent studies have found that antibodies that enhance infection *in vitro* protect mice and macaques from SARS-CoV-2 infection *in vivo* (Li et al., 2021).

In conclusion, our findings could support the evaluation of vaccination strategies for recovered COVID-19 patients in the face of newly emerging variants, facilitate the assessment of the risk of disease progression in confirmed COVID-19 patients at an early stage of the disease, and clarify the risk of SARS-CoV-2 ADE.

## Data Availability Statement

The raw data supporting the conclusions of this article will be made available by the authors, without undue reservation.

## Ethics Statement

The studies involving human participants were reviewed and approved by TSGHIRB No. C202005067. The patients/participants provided their written informed consent to participate in this study.

## Author Contributions

C-HC, F-YC, L-CY, C-LL, and K-CC designed the study; C-HC, Y-HC, K-CC, and P-LT performed experiments; all authors analyzed data; K-CC, T-WC and L-CY wrote the manuscript with help from all authors. All authors contributed to the article and approved the submitted version.

## Funding

This research was funded by grants from the Ministry of Science and Technology, Taiwan (MOST 109-2320-B-016-012 to L-CY, MOST 109-2327-B-016-004 and MOST 110-2327-B-016-001 to F-YC), and Ministry of National Defense Medical Affairs Bureau (MAB-110-104 to L-CY).

## Conflict of Interest

The authors declare that the research was conducted in the absence of any commercial or financial relationships that could be construed as a potential conflict of interest.

## Publisher’s Note

All claims expressed in this article are solely those of the authors and do not necessarily represent those of their affiliated organizations, or those of the publisher, the editors and the reviewers. Any product that may be evaluated in this article, or claim that may be made by its manufacturer, is not guaranteed or endorsed by the publisher.
