# Supplementary figures and images for: Humoral, Cellular and Cytokine Immune Responses Against SARS-CoV-2 Variants in COVID-19 Convalescent and Confirmed Patients With Different Disease Severities
Source: Front Cell Infect Microbiol. 2022 May 17;12:862656. doi: 10.3389/fcimb.2022.862656 (PMC9152113; doi:10.3389/fcimb.2022.862656)

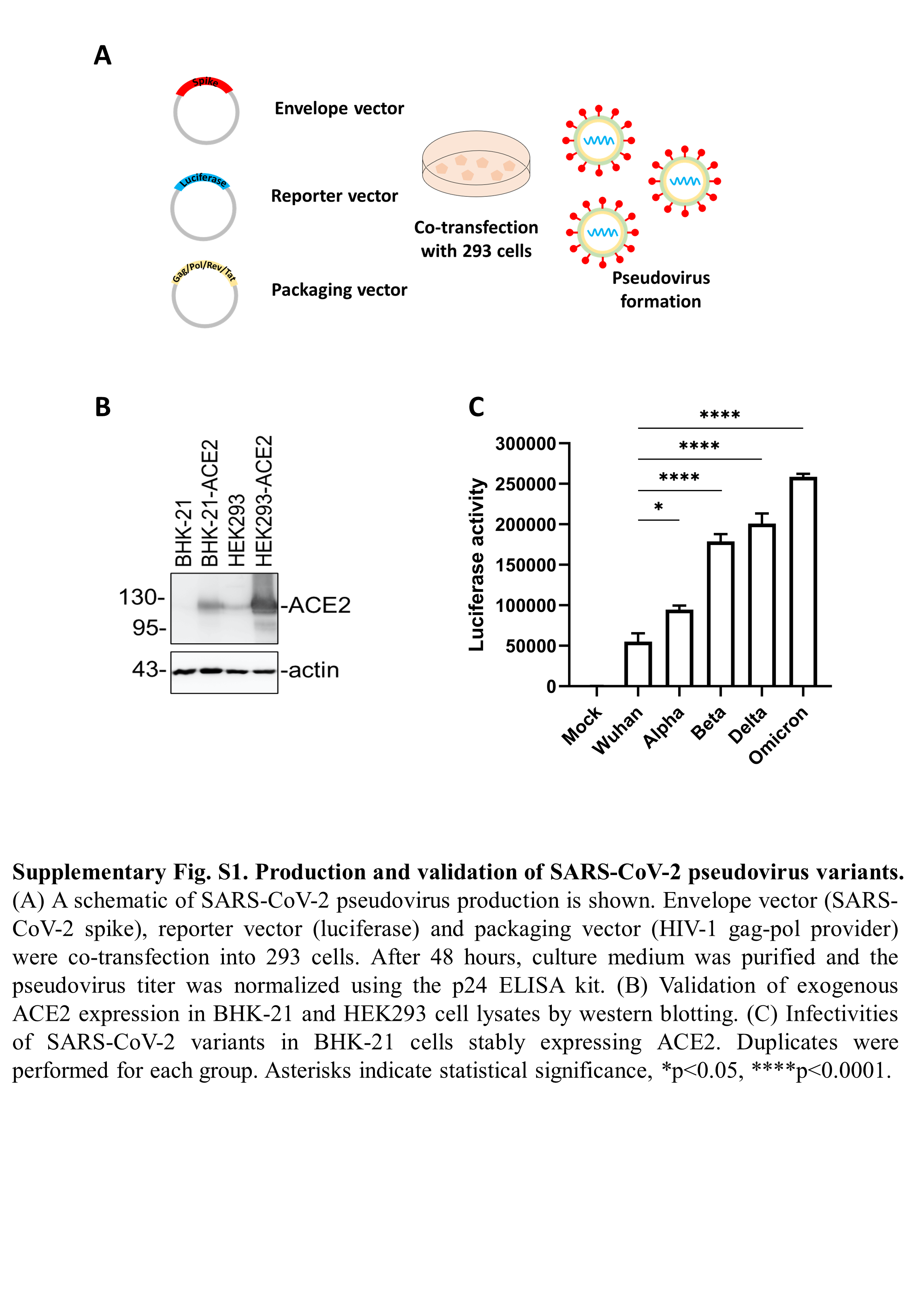

Supplement: Supplementary file 1 [file Image_1.tif]

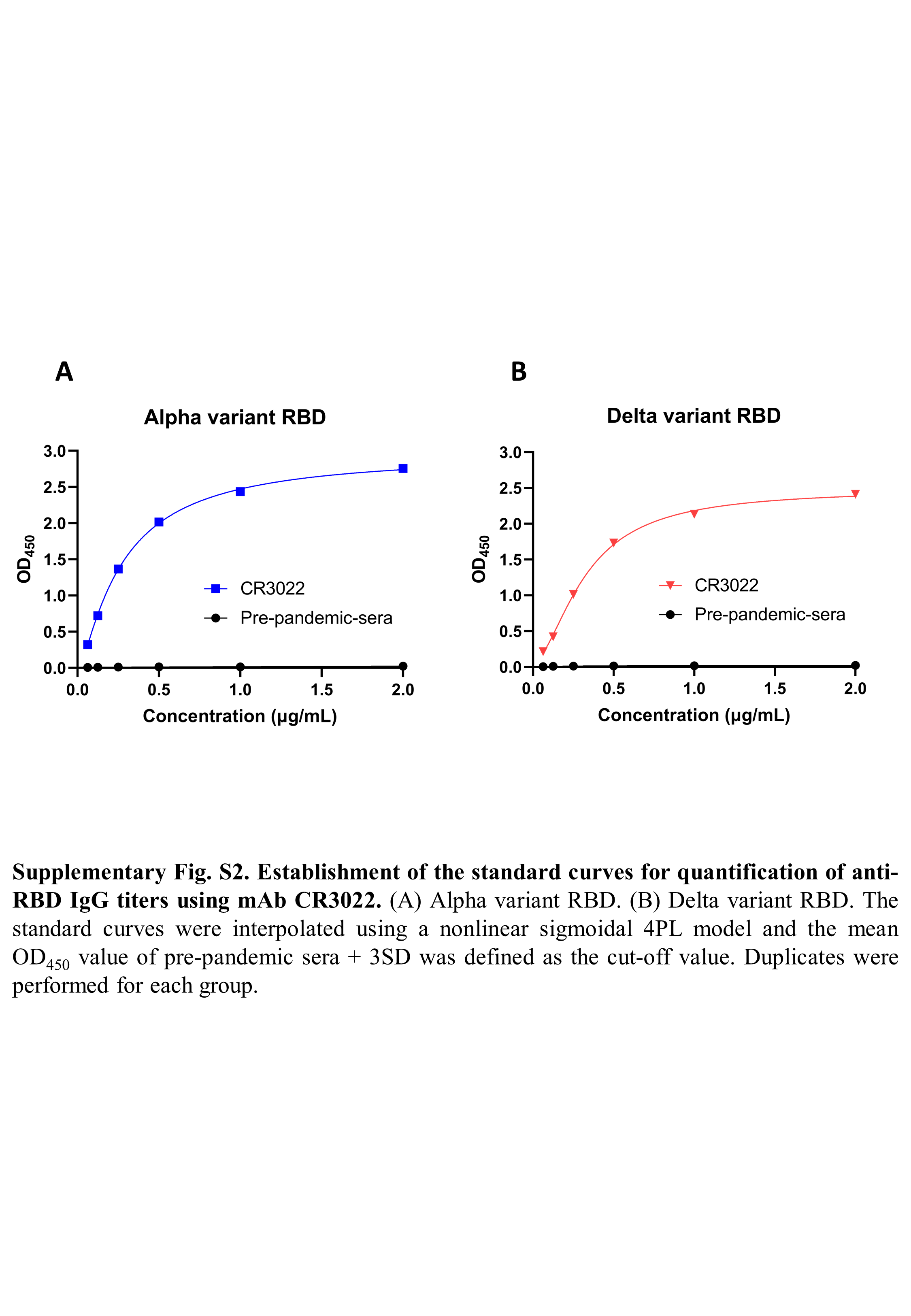

Supplement: Supplementary file 2 [file Image_2.tif]
