# Supplementary material for: Humoral, Cellular and Cytokine Immune Responses Against SARS-CoV-2 Variants in COVID-19 Convalescent and Confirmed Patients With Different Disease Severities
Source: Front Cell Infect Microbiol. 2022 May 17;12:862656. doi: 10.3389/fcimb.2022.862656 (PMC9152113; doi:10.3389/fcimb.2022.862656)
Supplement: Supplementary file 3 [file DataSheet_1.docx]

| Gender | Age | Underlying disease | Time of infection | Time of sampling | Time of discharge | Disease severity | Symptoms |
| --- | --- | --- | --- | --- | --- | --- | --- |
| M | 35 | Bronchial asthma | 2021.6.9 | 2021.8.3 | 2021.7.5 | Severe | fever, headache, dyspnea |
| F | 26 |  | 2021.6.10 | 2021.7.25 | 2021.6.23 | Mild | altered sense of taste and smell |
| M | 57 | Diabetes | 2021.5.28 | 2021.7.19 | 2021.6.13 | Severe | fever, dyspnea |
| M | 77 | Diabetes, Hypertension | 2021.5.23 | 2021.7.24 | 2021.6.27 | Severe | fever, dyspnea, cough |
| F | 75 | Diabetes, Old CVA | 2021.5.29 | 2021.8.10 | 2021.7.13 | Severe | fever, dyspnea, cough |
| M | 70 | Hypertension | 2021.5.24 | 2021.8.23 | 2021.7.28 | Severe | fever, dyspnea |
| M | 64 | Diabetes, Hypertension | 2021.6.2 | 2021.8.5 | 2021.7.9 | Severe | fever, diarrhea, dyspnea |
| M | 54 | Hypertension | 2021.5.30 | 2021.8.8 | 2021.7.11 | Severe | fever, dyspnea, cough |
| F | 58 |  | 2021.5.14 | 2021.7.8 | 2021.6.2 | Mild | fever, sorethroat, general malaise |
| M | 59 |  | 2021.5.24 | 2021.7.12 | 2021.6.15 | Severe | cough, dyspnea, chest pain |
| M | 70 | Hypertension, Hyperlipidemia | 2021.5.22 | 2021.7.26 | 2021.6.28 | Severe | fever, cough, dyspnea |
| F | 79 | Hypertension | 2021.5.20 | 2021.7.28 | 2021.6.29 | Severe | fever, cough, dyspnea |
| F | 61 | Chronic hepatitis B | 2021.5.23 | 2021.7.21 | 2021.6.18 | Mild | sorethroat, runny Nose |
| M | 57 |  | 2021.5.30 | 2021.8.8 | 2021.7.5 | Severe | fever, cough, dyspnea |
| F | 54 | Chronic urticaria | 2021.6.17 | 2021.8.10 | 2021.7.6 | Severe | fever, headache, dyspnea, chest tightness |
| F | 72 | Coronary artery disease, Parkinsonism | 2021.5.15 | 2021.6.28 | 2021.5.30 | Severe | fever, dyspnea, chest tightness |
| F | 35 |  | 2021.5.12 | 2021.6.26 | 2021.5.23 | Mild | sorethroat, runny Nose |
| F | 37 |  | 2021.5.10 | 2021.6.27 | 2021.5.23 | Mild | No symptoms |
| F | 93 | UCC of bladder, Atrial fibrillation, Chronic respiratory failure | 2021.5.30 | 2021.8.4 | 2021.7.8 | Severe | fever, dyspnea |
| M | 45 |  | 2021.5.26 | 2021.7.17 | 2021.6.14 | Mild | runny Nose, diarrhea |
| M | 58 | Coronary artery disease, Cirrhosis of liver, Valvular heart disease | 2021.7.18 | 2021.8.25 | 2021.7.29 | Mild | No symptoms |
| F | 54 | Coronary artery disease | 2021.7.18 | 2021.9.1 | 2021.8.3 | Mild | fever, chest tightness |
| M | 23 |  | 2021.7.9 | 2021.8.18 | 2021.7.20 | Mild | altered sense of taste and smell |
| F | 69 | Hypertension | 2021.5.31 | 2021.7.8 | 2021.6.11 | Severe | fever, dyspnea |
| M | 67 | Hypertension, Hyperlipidemia | 2021.5.28 | 2021.8.27 | 2021.7.30 | Severe | fever, dyspnea |
| M | 54 |  | 2021.5.31 | 2021.8.14 | 2021.7.16 | Severe | fever, dyspnea, cough |

Supplementary Table 1: Clinical characteristic of alpha-convalescent patients.

M: Male, F: Female, UCC: Urothelial cell carcinoma, CVA: Cerebrovascular accident.

| Gender | Age | Underlying disease | Time of infection | Time of sampling | Time of discharge | Disease severity | Symptoms |
| --- | --- | --- | --- | --- | --- | --- | --- |
| M | 45 | Coronary artery disease, Epilepsy | 2021.5.24 | 2021.6.9 | 2021.6.20 | Mild | No symptoms |
| M | 25 | Bronchial asthma | 2021.6.11 | 2021.6.30 | 2021.7.7 | Severe | Fever, Headache, Dyspnea |
| F | 58 |  | 2021.5.15 | 2021.6.9 | 2021.6.18 | Mild | Headache, Cough, Runny nose |
| F | 24 |  | 2021.6.11 | 2021.7.6 | 2021.7.11 | Mild | Altered sense of taste and smell |
| M | 53 | Cirrhosis of liver | 2021.6.1 | 2021.6.26 | 2021.7.16 | Severe | Fever, Dyspnea |
| M | 74 | Hypertension | 2021.5.26 | 2021.6.21 | 2021.7.21 | Severe | fever, dyspnea, cough |
| F | 78 | Diabetes, Hypertension, old CVA | 2021.5.30 | 2021.6.22 | 2021.7.23 | Severe | fever, dyspnea |
| M | 67 | Hypertension | 2021.5.28 | 2021.6.17 | 2021.9.2 | Severe | fever, dyspnea |
| M | 62 | Diabetes, Hyperlipidemia | 2021.6.3 | 2021.6.30 | 2021.10.5 | Severe | fever, diarrhea, dyspnea |
| M | 54 |  | 2021.5.31 | 2021.6.29 | 2021.7.29 | Severe | fever, dyspnea, cough |
| M | 69 |  | 2021.8.2 | 2021.8.14 | 2021.8.18 | Mild | No symptoms |
| M | 38 |  | 2021.6.4 | 2021.6.11 | 2021.7.3 | Mild | cough, sorethroat, headache |
| M | 42 |  | 2021.6.6 | 2021.6.17 | 2021.7.3 | Mild | cough, sorethroat, runny Nose |
| F | 65 |  | 2021.5.18 | 2021.6.13 | 2021.7.3 | Severe | fever, cough, diarrhea |
| M | 77 | Hypertension | 2021.6.3 | 2021.6.30 | 2021.8.26 | Severe | fever, cough, dyspnea |
| F | 64 | Diabetes, Schizophrenia | 2021.6.2 | 2021.6.17 | 2021.6.25 | Severe | fever, dyspnea |
| M | 39 | Seizure | 2021.5.26 | 2021.6.17 | 2021.6.29 | Mild | fever, dyspnea, cough |
| M | 31 |  | 2021.6.21 | 2021.7.8 | 2021.7.18 | Mild | fever, sorethroat, myalgia |
| M | 73 | Diabetes, end stage renal disease | 2021.6.5 | 2021.7.2 | 2021.7.16 | Severe | fever, dyspnea |
| M | 68 |  | 2021.6.4 | 2021.6.14 | 2021.6.19 | Mild | No symptoms |
| M | 30 |  | 2021.6.5 | 2021.6.13 | 2021.7.3 | Mild | cough, runny Nose |
| M | 37 |  | 2021.6.7 | 2021.6.17 | 2021.7.3 | Mild | No symptoms |

Supplementary Table 2: Clinical characteristics of alpha-confirmed patients.

M: Male, F: Female, CVA: Cerebrovascular accident.
